# Make Better Choices (MBC): Study design of a randomized controlled trial testing optimal technology-supported change in multiple diet and physical activity risk behaviors

**DOI:** 10.1186/1471-2458-10-586

**Published:** 2010-09-29

**Authors:** Bonnie Spring, Kristin Schneider, HG McFadden, Jocelyn Vaughn, Andrea T Kozak, Malaina Smith, Arlen C Moller, Leonard Epstein, Stephanie W Russell, Andrew DeMott, Donald Hedeker

**Affiliations:** 1 Department of Preventive Medicine, Northwestern University, Chicago, Illinois, USA; 2 Department of Psychology, University of Illinois - Chicago, Chicago, Illinois, USA; 3 Department of Social and Preventive Medicine, University of Buffalo, Buffalo, New York, USA; 4 Department of Epidemiology and Biostatistics, University of Illinois - Chicago, Chicago, Illinois, USA

## Abstract

**Background:**

Suboptimal diet and physical inactivity are prevalent, co-occurring chronic disease risk factors, yet little is known about how to maximize multiple risk behavior change. Make Better Choices, a randomized controlled trial, tests competing hypotheses about the optimal way to promote healthy change in four bundled risk behaviors: high saturated fat intake, low fruit and vegetable intake, low physical activity, and high sedentary leisure screen time. The study aim is to determine which combination of two behavior change goals - one dietary, one activity - yields greatest overall healthy lifestyle change.

**Methods/Design:**

Adults (n = 200) with poor quality diet and sedentary lifestyle will be recruited and screened for study eligibility. Participants will be trained to record their diet and activities onto a personal data assistant, and use it to complete two weeks of baseline. Those who continue to show all four risk behaviors after baseline recording will be randomized to one of four behavior change prescriptions: 1) increase fruits and vegetables and increase physical activity, 2) decrease saturated fat and increase physical activity, 3) increase fruits and vegetable and decrease saturated fat, or 4) decrease saturated fat and decrease sedentary activity. They will use decision support feedback on the personal digital assistant and receive counseling from a coach to alter their diet and activity during a 3-week prescription period when payment is contingent upon meeting behavior change goals. They will continue recording on an intermittent schedule during a 4.5-month maintenance period when payment is not contingent upon goal attainment. The primary outcome is overall healthy lifestyle change, aggregated across all four risk behaviors.

**Discussion:**

The Make Better Choices trial tests a disseminable lifestyle intervention supported by handheld technology. Findings will fill a gap in knowledge about optimal goal prescription to facilitate simultaneous diet and activity change. Results will shed light on which goal prescription maximizes healthful lifestyle change.

**Trial Registration:**

Clinical Trials Gov. Identifier NCT00113672

## Background

Poor quality diet and physical inactivity are well-established behavioral risk factors for heart disease, cancer, and diabetes [1-3]. Healthy lifestyle change can reduce morbidity and premature mortality [4,5]. However, fewer than 25% of U.S. adults have lifestyles that meet dietary or physical activity guidelines [6-8]. Unhealthy diet and physical inactivity represent particularly deserving targets for intervention because they represent major health risk factors for a majority of Americans.

Suboptimal diet and a predominately sedentary behavior pattern illustrate what we call *multi-behavioral morbidity*: a co-occurrence of behavioral pathogens also characterized as *risk behavior bundling*. These and other unhealthy lifestyle behaviors overlap frequently in the same individuals [9-11], heightening disease risk [12]. Behavior bundling creates an opportunity to intervene comprehensively, efficiently, and perhaps even synergistically on more than one risk behavior simultaneously. What remains unknown for adult populations, though, is how to frame behavioral prescriptions so as to optimize healthy change in multiple risk behaviors simultaneously. An important gap in knowledge concerns whether it is more advantageous to increase low rate healthy behaviors, decrease high rate unhealthy behaviors, or change some combination of the two. The Make Better Choices (MBC) study is a comparative clinical trial designed to discern an optimal approach to simultaneous diet and activity change. MBC compares four different intervention approaches to changing a portfolio of two dietary risk behaviors--low fruit and vegetable intake and high saturated fat consumption--and two activity risk behaviors--low moderate-vigorous intensity physical activity and high sedentary leisure screen time.

### Three Alternative Hypotheses

The primary aim of the MBC trial is to test three alternative hypotheses about which behavioral prescription maximizes overall healthy lifestyle change: Familiarity, Behavioral Economic, or Low Inhibitory Demand. We describe each hypothesis and its rationale in turn.

According to the *familiarity hypothesis*, the most effective behavior change prescription will be the one most familiar based upon usual experience with trying to lose weight. If this hypothesis is supported, the most efficacious intervention will be the customary dieting approach that prescribes decreasing saturated fat and increasing physical activity.

An alternative *behavioral economic hypothesis *predicts that the most efficacious regimen will be the one that maximizes healthy substitutive or complementary behavior change in both targeted and collateral behaviors. Healthy substitution comes about when a targeted healthy behavior replaces (crowds out) an untargeted unhealthy one. Healthy complementary (tag-along) change occurs when a targeted increase in a healthy behavior is accompanied by an untargeted increase in a low-rate healthy behavior, or when a targeted decrease in an unhealthy behavior brings along a decline in an untargeted unhealthy behavior. Behavioral economic theory holds that some concurrently available reinforcers can interact with each other as substitutes such that as consumption of one decreases (due to increased cost or reduced availability), consumption of the other increases [13]. To the extent that physical activity is substitutable for sedentary leisure activity, physical activity should increase as sedentary leisure time is scaled back.

Findings from studies with children indicate that healthful substitution occurs, at approximately a 1:3 ratio of physical activity gain versus inactivity loss [14]. To our knowledge, no comparable data exist for adults. To the extent that fruits and vegetables are substitutable for high saturated fat foods, increasing their consumption should reduce the intake of saturated fat. For children, fruit and vegetables do not appear to substitute for high fat foods [15,16]. However, it is unknown whether greater substitutability occurs for adults. Behavioral economic theory would predict that the prescription to increase fruit and vegetable intake while decreasing sedentary leisure behaviors should result in the best overall dietary and activity change. By optimal substitution, eating more fruits and vegetables should lower the intake of high saturated fat foods and reducing time spent on sedentary leisure activities should allow physical activity to increase. By optimal complementarity, increasing physical activity should foster eating more fruits and vegetables, and reducing sedentary activity should also reduce intake of saturated fat.

Finally, the *low inhibitory demand hypothesis*, consistent with self-regulatory strength theory, posits that the best approach to improve eating and activity simultaneously is one that minimizes demands on participants' limited self-regulatory resources by not requiring inhibition of overlearned, rewarding, consummatory responses. Research findings suggest that exerting self-control by inhibiting responses to temptation depletes a limited resource, reducing the strength available for further control efforts [17]. More taxing self-regulation tasks deplete resources more extensively, as evidenced by the onset of self-regulatory fatigue or ego-depletion [18]. Making demands on self-control also lessens the degree to which highly overlearned reward self-administration behaviors (e.g., eating saturated fats, watching television) can continue to be suppressed, heightening vulnerability to temptations to abandon self-control. In particular, prior research has demonstrated that avoidance-oriented forms of self-regulation that require inhibition (e.g., avoiding saturated fats, avoiding sedentary activity) are particularly debilitating [19]. Thus, the *low inhibitory demand hypothesis *predicts that increasing fruits and vegetables while increasing physical activity will be the most successful prescription because this approach places the least demand on inhibitory self-control.

### Leveraging Handheld Technology

**Figure 1:**
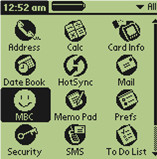
**Screen shot of the MBC program on a PDA**. Participants select the MBC icon to open the program.

**Figure 2:**
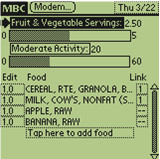
**Screen shot of the goal thermometers**. The goal thermometers reflect the fruit and vegetable consumption and moderate activity of a hypothetical participant.

The MBC study will also examine the feasibility of incorporating a personal digital assistant (PDA)-based decision support tool to encourage multiple health behavior change. The PDA's compactness and portability enables participants to carry the device and record eating and activity immediately after completing a behavior. Figure 1 shows a screen shot of the MBC program on a PDA. Goal "thermometers" on the PDA provide 'in the moment' decision support to participants by continually displaying their progress relative to prescribed daily diet and activity goals. The PDA decision support tools also make it feasible for participants to explore and anticipate the consequences for goal attainment if they were to engage in potential eating or activity behaviors. A screen shot illustrating the PDA thermometers is shown in Figure 2. Daily electronic transmission of time-stamped diet and activity data from the PDA to a coach fosters accountability and enables distance support provision from a coach via telephone or e-mail (per participant preference). Coaching algorithms suggest low saturated fat substitutes and liked, but rarely chosen vegetables or physical activities, enabling paraprofessionals to serve as behavioral coaches.

## Methods/Design

### Study Design

The research design is a four by three factorial involving one between subjects factor (condition) with four levels and one within subjects factor (time) with three levels (baseline, prescription, follow-up). Inactive adults with suboptimal diet (N = 200) will be randomly assigned to one of four treatment conditions: a) ↑FV↑PA increase fruit and vegetable consumption to >5/day and increase moderate-vigorous physical activity to >60 minutes/day); b) ↓Fat ↓Sed: decrease saturated fat consumption to < 8% per day and decrease targeted sedentary leisure activity to < 90 minutes/day); c) ↑FV↓Sed: increase FV consumption to > 5/day and decrease Sed to < 90 min/day); or d) ↓Fat↑PA decrease saturated fat to < 8%/day and increase PA to > 60 min/day). The four intervention conditions represent a complete crossing of approach and avoidance oriented goals related to diet (↑FV or ↓Fat) and activity (↑PA or ↓Sed).

**Figure 3:**
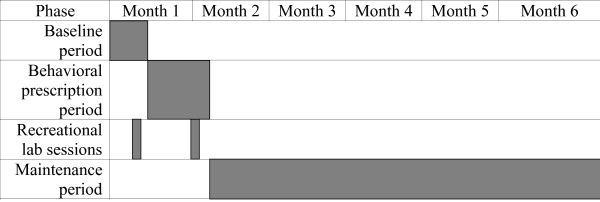
**Protocol timeline**. Participants record diet and activity daily for 2 weeks of baseline, followed by 3 weeks of behavioral prescription. They complete one recreational laboratory session during baseline and one during prescription. They record intermittently during 4.5 months of follow-up after prescription period ends.

Participants will be studied through three study phases: a) usual behavior baseline (2 weeks); b) prescription phase (3 weeks) during which participants' payment will be contingent upon meeting pre-specified behavior change standards; and c) follow-up phase (4.5 months) during which payment will be contingent upon behavioral recording only. The protocol timeline is shown in Figure 3.

#### Recruitment and Screening Process

The study procedures are approved by the Institutional Review Boards at the University of Illinois at Chicago and Northwestern University. Community-dwelling adults between ages 21 and 60 will be recruited via flyers, newspaper advertisements and e-mails to staff affiliated with these two large urban universities. Recruitment materials will seek "couch potatoes" needed for a paid research study. Interested candidates will be directed to a website that explains the study and prompts completion of a consent process and on-line screening questionnaires. Screening instruments assess for low fruit and vegetable consumption and high saturated fat intake [20], high sedentary leisure behavior, and low physical activity [21]. To qualify as eligible, participants must report all of the following: a) < 5 FV/day; b) >8% saturated fat intake; c) < 60 min/day moderate-vigorous PA; and d) > 90 min/day targeted Sed. Sedentary activity was defined conservatively as engagement in optional, recreational "screen-time" activities: specifically, television, movies, non-work related computer use, and video games.

Further eligibility screening will be performed by telephone interview and then in-person screening. For safety, participants with unstable medical conditions (e.g. uncontrolled hypertension, recent myocardial infarction), current eating or substance abuse disorder (besides tobacco use), current suicidal ideation, or weighing more than 300 lb. will be excluded. Those with stable medical conditions may enroll with physician approval to participate. To minimize risk associated with increasing physical activity, participants must be able to complete the Dundee 3-minute step test [22] and sustain a moderate walking pace on a treadmill during in-person screening. Individuals with Celiac disease, practicing severe dietary restrictions, or involved in a formal diet or activity program will be excluded. Females may not be pregnant, trying to get pregnant, or lactating. Study candidates must have enough scheduling flexibility to be able to complete two three-hour laboratory sessions that are part of the study protocol. Finally, in order to be able to transmit PDA data, participants require either a home computer with internet access or a landline telephone for use with a loaned modem.

#### Baseline Phase (and final eligibility screening)

Participants who pass initial screening will undergo an informed consent process with the Bachelor's level research assistant who will serve as the person's coach if eligibility is confirmed. The coach will perform an equipoise induction balancing the desirability of the four intervention conditions. He or she will assess anthropometric and demographic information and will dispense a PDA and train the individual on its use. Coaches will train participants to accurately estimate dietary intake. They will instruct participants to recognize moderate intensity physical activity by providing feedback about their heart rate (level of exertion) as they walk at gradually increasing speeds on a treadmill. For a two-week baseline period, participants will wear an accelerometer and use the PDA to record diet and activity, and upload data daily to their coach.

#### PDA Tool

The PDA will be pre-loaded with a version of CyberSoft's Nutribase as well as a modified Compendium of Physical Activities [23] and software designed for the study. Cybersoft's nutrient database contains nutrient data for more than 30,000 foods. The Physical Activity Compendium has been expanded to include additional sedentary activities. A user-friendly interface enables participants to: a) click drop-down menus to enter foods and activities, b) conduct text searches for foods and activities, c) link entries to convey items eaten together as a combined food (e.g., sandwich combining bread, tomato, bacon, lettuce, mayonnaise), and d) enter and store custom foods. The software date- and time-stamps participants' entries and displays them to the coach in an organized format on the study server. Participants are instructed to enter foods immediately after eating them. Food entry prompts a "pop-up" question that, depending upon study condition, inquires about minutes of physical and/or targeted sedentary behavior since the last eating episode.

Coaches will provide corrective feedback when implausible reports suggest inaccurate or untimely reporting. Enrollees whose PDA and accelerometer data indicate that they display all four behavioral risk factors throughout the baseline period will be randomized. (This selection policy retains only high risk cases whose behavioral morbidities fail to remit spontaneously.) Participant allocation to condition will be concealed until the start of prescription period, when the project statistician determines the random assignment. Randomization, stratified by gender, will be performed using a computer generated sequence and the method of randomly permuted blocks.

#### Behavioral Prescription Period

An individual meeting between the coach and participant will begin the 3-week behavioral prescription phase. During the in-person session, the coach will convey the randomized treatment assignment and provide behavioral coaching. The session will begin by providing participants with a report detailing their to-be-targeted dietary intake and activity behaviors during the 2-week baseline period. Coaches and participants will engage in shared decision-making to formulate specific tailored behavioral changes (four diet and four activity) to help participants reach their goals. They also will identify barriers to engaging in healthy lifestyle behaviors and will problem-solve solutions. To increase fruits and vegetables or physical activities, the prescriptions will highlight liked but low rate FVs and PAs identified during screening and will problem-solve about how to increase these. To decrease Fat and Sed, coaches will review the foods that contributed the greatest saturated fat and the recreational screen time activities that consumed the most time during baseline. They will strategize with participants about lower fat alternatives and non-screen-time recreational sedentary activities (e.g., reading, listening to music, conversation) that can be substituted. Coaches will exercise treatment fidelity by taking particular care to recommend only those behavior changes prescribed by the assigned treatment condition. For example, for participants randomized to decrease sedentary pastimes, the coach will refrain from advising an increase in physical activity. Likewise, for those randomized to decrease foods high in saturated fat, the coach will avoid suggesting increased consumption of fruits and vegetables.

The coach will activate two feedback and decision support thermometers on the participant's PDA - one for diet and one for activity. Depending on participant's assigned condition, the diet thermometer will display either what percent of the day's FV intake goal has been attained thus far or how much of the day's saturated fat gram allowance has been used. Similarly, the activity thermometer will display either how much of the day's physical activity goal has been attained thus far or how much of the day's targeted recreational sedentary activity allowance has been used. For prescription week 1, goals will be set at the half-way point: a level of attainment that is midway between the individual's baseline diet and activity levels and the prescribed target levels. Goals and goal thermometers will be re-set for prescription weeks 2 and 3 to the full target goal standard. Participants will continue to upload data daily during all three weeks of the prescription phase. They will communicate with their coaches via telephone or e-mail to problem-solve challenges with attainment of behavioral targets. The coach will also help participants troubleshoot any difficulties with study equipment and will, as needed, call in technical support.

Consistent with 5-A-Day Program Guidelines [24], the PDA program assigns participants no credit for FVs that have a high calorie content (e.g., avocados, coconuts, olives). FVs with added fat and sodium will also be excluded if more than 30% of calories are from fat, more than 10% of calories are from saturated fat, or more than 480 milligrams of sodium have been added. FVs that are processed by drying, freezing, or canning will be included, as will any juice that is 100% juice or juice concentrate. A serving will be defined by USDA [25] guidelines as: a medium piece of fruit; half a cup of raw, cooked, canned, or frozen FVs; one cup of leafy salad greens; one quarter cup of dried fruit; six ounces (three-quarters cup) of 100% fruit or vegetable juice; or a half cup of cooked or canned beans, peas, or legumes (e.g., lentils, black beans).

#### Recreational Laboratory Sessions

Half-way through the baseline period and again two weeks into prescription phase, participants will attend a 4 hour recreational laboratory session. The purpose of the recreational lab sessions is to measure predictors and hypothesized mediators of the intervention effects. Laboratory sessions will be held during lunch or dinner time (11:00 am to 9:00 pm). The sessions will be framed for participants as a brief break from needing to monitor food intake and activity or follow a behavioral prescription. Participants will be advised to wear comfortable clothing and to expect to eat a meal and snacks and to engage in sedentary and/or physical activity of their own choosing. Upon arrival, participants will be asked to provide a urine sample. They will be told that the sample will be assayed to bioverify their dietary intake, a bogus pipeline cover story designed to increase the veridicality of participants' dietary reports. Then they will complete a battery of questionnaires (described below), a modified Stroop Test designed to measure attentional bias [26], and a Concurrent Schedules Choice Task designated as the Want Task.

In the Want Task, participants will use study funds to purchase pre-weighed portions of FVs and high saturated foods and minutes of sedentary and physical activities that they can access during their upcoming time in the recreational laboratory. The foods and activities made available for purchase are pre-determined to be options that the participant enjoys. Participants know the quantity units available for purchase, understand that the commodities they purchase cannot be taken home, and know that they will be the only foods and activities available during the three-hour recreational laboratory session that follows the Want Task.

When the task is finished, research staff will tally the amount of each commodity purchased and will stock the lab accordingly. The lab is equipped with a refrigerator, sink, microwave, table and chairs, couch, computer station, cable television and DVDs, exercise bike, treadmill, exercise mat, weights, exercise ball and bands. Two timing devices will be set: one for minutes of PA purchased; the other for minutes of Sed, so that minutes used can be tabulated against those activity allotments. When not eating or engaging in purchased PA or Sed, participants will be able to read the daily newspaper at no charge. The 3-hour laboratory session will be observed and videotaped remotely. Plates containing purchased high-saturated fat meal and snack items and fruits and vegetables will be weighed prior to the session and at the session's end. Plate waste will be calculated to measure consumption. Trained research specialists will code the videotapes for time spent on targeted Sed, PA, and eating, and for whether the activities were performed singly or in combination.

#### Follow-Up Period

Once the prescription period ends, decision support thermometers will remain visible on the PDA, but participants will be informed that adherence to behavioral goal targets is no longer required to receive compensation. Payment will now be contingent only upon recording and transmitting PDA data on a predetermined schedule. Participants will record diet and activity daily for the first week following prescription. Subsequent recording periods will be three consecutive days long. They will occur weekly for the second and third weeks after prescription, biweekly for the next six weeks, and monthly for the final eight weeks. At a debriefing session after the follow-up period, the bogus pipeline will be unmasked.

#### Financial Incentives

To encourage retention and initial goal attainment, participants will earn the following financial incentives for successfully completing each phase of the trial: Screening - $20; Lab 1- $20; Baseline week 1 - $20; Baseline week 2 - $20; Prescription - $175; Follow up 1 - $50; Follow up 2 - $30; Follow up 3 - $35; Follow up 4 - $40; Follow up 5 - $45; Follow up 6 - $50; Follow up 7 - $55; Follow up 8 - $80; Full compliance bonus - $50, yielding maximum total payment of $730. All financial incentives will be offered solely for completing assessments, except for the prescription phase incentive, which requires also meeting behavioral targets, and the bonus payment, which requires completing all assessments on time. Payment will be cumulated over the course of study participation and paid out at termination when the participant returns the PDA.

### Assessment Instruments

#### Screening

MBC's web-based dietary screening questionnaire will be the 27-item Rapid Food Screener [27], a food frequency questionnaire that estimates daily fruit/vegetable servings, total fat, saturated fat, and percent total and saturated fat. The web-based activity screener will be the Physical Activity Recall Questionnaire (PAR) [21], an 8-item questionnaire that assesses moderate-vigorous activity for the past 7 days and past month. Because no validated screening measure was available for sedentary screen time, we created a parallel 5-item questionnaire to assess time devoted to targeted sedentary activities over the same period as the PAR. Current substance abuse, eating disorders, and suicidality will be evaluated by the Structured Clinical Interview for DSM-IV Diagnosis--Non-Patient Version (SCID-NP) [28] and the Hamilton Rating Scale for Depression [29] during the in-person screening visit.

#### Descriptive

Demographic information, anthropometric data, motivation and readiness for health behavior change, and past history of dieting and exercise will be assessed during screening. Demographic data to be gathered are: gender, age, ethnicity, marital status, education, income, and household size. Weight and height will be measured using a balance-beam scale to the nearest 1 lb and .1 in, respectively. Body Mass Index (BMI) will be calculated as (weight in pounds/(height in inches) 2) × 704.5, the mathematical equivalent of the BMI metric calculation (kg/m2). Waist and hip circumference will be assessed using a tape measure, to the nearest .1 in.

Reasons for enrolling in the study will be measured by a modified Motivation for Physical Activities scale [30]. Items have been altered to ask about eating as well as activity changes. Seven questions about financial motivation to participate have been added, and the social scale has been deleted (as not applicable). The resulting 32-item Motivation for Health Behavior Change in an Experimental Setting questionnaire yields five motive subscales (fitness, appearance, competence/challenge, enjoyment, and financial incentives). To assess prior dieting experience, participants will complete a four-item Diet Familiarity Scale adapted from Project EAT [31]. The Familiarity Scale asks about previous experience with each prescribed eating and activity change. To examine the influence of response bias on diet and activity self-reports, the degree of felt need to present oneself in a favorable light will be assessed during screening by the 33-item Marlow-Crowne Social Desirability Scale [32].

Activity Liking and Craving Questionnaires will be administered during screening as an aid for coaching and a means to identify preferred foods and activities to be offered during recreational laboratory sessions. The questionnaires assess self-reported preference and urge to consume specific FV, high saturated fat foods, physical activity, and sedentary leisure activities. Each questionnaire presents familiar items that participants rate using Rozin et al's (1991) [33] 9-point hedonic scale. Participants will complete the questionnaires once to express food and activity liking, where response options range from 1 = dislike extremely to 9 = like extremely. Then, without access to their liking ratings, they will re-rate the items to report craving. Anchors for the 9-point craving scale will range from 1 = little or no urge to 9 = extreme urge. The Food Liking and Craving Questionnaire presents 10 sweet foods high in saturated fat, 10 savory foods high in saturated fat, 10 vegetables, and 10 fruits. The Activity Liking and Craving Questionnaire presents 10 moderate/vigorous intensity athletic activities, 10 moderate intensity work or household activities, 10 sedentary work or home-related activities, and 10 sedentary recreational activities including the ones targeted for change in this study. Coaches will use these measures to identify low rate but liked FV and activities to suggest to participants. Participants' responses will also be used to select four fruits/vegetables and four physical activities with liking ratings of approximately 5 and four high saturated foods and four sedentary pastimes with ratings of approximately 7. Those items will be offered for purchase in the Want Task and made available in the recreational laboratory. Participants will rate their liking for the eight chosen reinforcers prior to each recreational laboratory session in order to verify that the options offered are experienced as pleasurable.

#### Psychological and Behavioral Process Measures

To identify psychological processes that accompany (and potentially mediate) behavior change, a series of measures will be administered towards the end of the baseline and prescription periods. Readiness and self-efficacy to make behavioral changes will be assessed during baseline and prescription periods. Readiness to change physical activity will be evaluated by Physical Activity Staging (PAS) [34]. Readiness to change sedentary leisure will be measured by a parallel PAS Sedentary Behavior Staging Questionnaire. Dietary readiness to change FV and Fat intake will be measured, respectively, by the Rhode Island Cancer Prevention Research Center's (1995) [35] Stage of Change for 5-A-Day and Stage of Change for Dietary Fat scales. Self-Efficacy to Increase Fruits and Vegetables and Decrease Saturated Fat [36] will also be measured, as will Self-Efficacy to Increase Exercise and Decrease Sedentary Behavior [37].

Changes in the attentional salience of healthy and unhealthy foods and activities will be measured by the Stroop Task given prior to both recreational laboratory sessions. The Stroop color-naming test quantifies the degree of interference produced by responding to words associated with high-fat foods, FVs, sedentary activity, and physical activity, as compared to neutral furniture words. A semantic category's ability to capture attention and produce response slowing or errors reflects its motivational salience. For example, attentional bias to food-related Stroop cards has been associated with food deprivation [38], intentional dietary restriction [39,40], and food craving [39].

The behavioral economic Want Task will be administered next at both laboratory sessions to measure the relative reinforcing value of healthy and unhealthy foods and activities. On each of the Want Task's 40 trials, participant will be given $4 to allocate across 15 g portions of food and 1 minute portions of activity. The relative price per unit of each commodity will vary: $0.25, $0.50, $1, $2, and $4. Participants will be advised to spend all of the money they are given, since none can be saved or taken home. Data will be scored to yield the following four outcome variables: 1) amount *purchased *of healthy/unhealthy commodities, 2) amount *consumed *of healthy/unhealthy commodities, 3) own-price elasticity/inelasticity (the degree to which participants' consumption of a good is influenced by price change for that good), and 4) cross-price elasticity/inelasticity (the degree to which participants' consumption of a good is influenced by price change for another good, resulting from substitution or complementarity).

Throughout the baseline and prescription phases participants will also report their mood and hunger. Daily, just after completing the end-of-day activity log, they will rate their hunger on a 1-10 scale and describe their mood using the Positive and Negative Affect Schedule (*PANAS*) [41].

### Primary Outcome Measures

#### Dietary Assessment

Saturated fat and FV consumption will be measured via daily PDA intake recordings. The saturated fat gram goal for those prescribed to decrease Fat intake will be determined by using the Harris-Benedict equation [42] to estimate number of calories needed to maintain weight. The saturated fat percentage then will be calculated using basal metabolic rate in order to prevent inflating the fat gram allowance due to superfluous calories (e.g. in beverages).

#### Activity Assessment

Physical activity will be measured by both accelerometry and self-report. For parallelism between the diet and activity outcomes, and between sedentary and physical activity assessments, the primary activity outcome will use self-report. During baseline, prescription, and follow-up periods, minutes of physical and sedentary activity will also be self-reported using an end-of-day 24-hour activity log in which participants account for every 15-minute time period of the day. To complete the log, participants will select activities from a modified version of the Compendium of Physical Activities [23], which defines moderate activity as any activity >3 METs. The Compendium will be altered for MBC by adding the four targeted sedentary leisure activities (i.e., television, movies, leisure computer, and video games). During baseline and prescription periods, minutes of moderate- and vigorous intensity physical activity also will be assessed directly using an accelerometer belted on the hip (Computer Science and Applications, Inc. Actigraph Model #7164).

#### Healthy Lifestyle Change

So that lifestyle behaviors are quantified on a common metric, we will generate z-scores reflecting each participant's average daily fruit/vegetable intake, saturated fat intake, daily physical activity, and daily targeted sedentary activity relative to the sample distribution at baseline. Z-scores will be standardized such that positive values indicate more healthful behavior change. Scores will be averaged across all four behaviors (FV, Fat, Sed, and PA) to derive a composite index that expresses the average healthy lifestyle change achieved by each participant.

### Analytic Plan

The primary analytic plan is designed to determine which behavioral prescription maximizes attainment of healthy lifestyle behavior change from baseline through prescription phase. The secondary analytic plan is to learn which prescription favorably influences behavioral and psychological processes, i.e., by fostering healthy substitutive or complementary change in lifestyle behaviors, fostering increased elasticity of unhealthy choices and increased inelasticity of healthy choices on the Want Task, and fostering increased self-efficacy, attentional bias, craving, and positive mood in association with healthy choices. The tertiary, exploratory, analytic plan is to examine maintenance of healthy lifestyle change. Distributions of all dependent variables will first be examined to search for outliers and to assess normality, with transformations undertaken as appropriate.

Outcomes will be evaluated within the context of three competing alternative hypotheses. The *familiarity hypothesis *posits that healthy simultaneous change in eating and activity will be maximized by the customary prescription to decrease intake of saturated fat and increase physical activity. The rationale is that this prescription reflects usual dieting and exercise practices, with which most adults have prior experience. The *behavioral economic *(optimal substitution-complementarity) *hypothesis *posits that healthy simultaneous change in eating and activity will be maximized when the prescription is to increase FVs while decreasing recreational screen time. The *low inhibitory demand hypothesis *posits that healthy simultaneous change in eating and activity will be maximized when the prescription requires no inhibition/suppression of any rewarding eating or activity behaviors. This hypothesis predicts that the prescription to increase FV and PA will most improve healthy lifestyle behaviors because it requires no inhibitory self-control over high-rate, pleasurable eating and activity behaviors.

#### Primary Aim - Healthy Lifestyle

The first aim is to test our three alternative hypotheses about the optimum way to advise people to make two healthy behavior changes simultaneously, one in eating and one in activity. For this, we will use a linear mixed model (LMM) for longitudinal data [43] treating the composite health lifestyle z-score as the dependent variable, and including prescription condition as a between subjects factor and time as a within-subjects factor. The hypotheses predict a condition × time interaction, indicating that some conditions are more effective than others at bringing about healthy behavior change. If the overall omnibus F test of the condition × time interaction is significant, three planned comparisons will be performed to test the three competing hypotheses following procedures recommended by Rosenthal, Roscoe, and Rubin (2000) [44]. These comparisons will contrast the groups based on change over time for the composite healthy lifestyle z-score. Additionally, if the condition × time interaction is significant for the composite z-score, we will follow-up the overall test with separate tests of the interaction effect on each health behavior.

#### Secondary Aim - Behavioral and Psychological Processes

The behavioral component of Aim 2 tests the same three alternative hypotheses about which prescription is superior to the others in terms of: a) increasing substitution of healthful for unhealthful eating and activity behaviors, b) promoting elasticity for unhealthful behaviors, and c) promoting inelasticity for healthy behaviors. Substitutability analyses will be performed on cross-consumption coefficients from PDA eating and activity data and behavioral economic laboratory data. Cross-price elasticities from the Want Task will additionally test whether those results match the ones derived from consumption data. Analysis of own price elasticities from the Want task will test the same three hypotheses regarding which prescription best promotes the desired healthier outcome: increased elasticity for sedentary behavior and saturated fat intake, and increased inelasticity for physical activity and fruit and vegetable intake. The form of these analyses will parallel those procedures specified for the primary aim.

The psychological component of Aim 2 tests which behavioral prescription has the most desirable effects on increasing attentional bias, craving, self-efficacy, and positive mood associated with healthy foods and activities, and decreasing those processes for unhealthy choices. As for the previous aims, these analyses will utilize a linear mixed model with prescription as the between subjects factor and time as the within-subjects factor, as described above.

#### Tertiary (Exploratory) Aim - Maintenance

The exploratory aim has two parts. Part A is to compare how well the healthy behavior changes produced by the different prescriptions maintain throughout four months of follow-up. The planned analyses will use mixed linear modeling with prescription condition as the between subjects factor and time (prescription weeks 1, 2-3, and follow-ups 1 through 8) as the within subjects factor. Part B is to examine whether the processes listed in Aims 2 and 3 (inelasticity, mood, attentional bias and craving for unhealthy eating and activities) mediate the trajectory toward relapse and account for differences between prescriptions in the behavioral decay curves they yield. Analyses for exploratory aim B will use Baron and Kenny's (1986) test of mediation. If the exploratory aim A analyses establish that prescriptions affect the rate at which healthy lifestyle gains decay, then regression analyses will test whether: a) condition affects behavioral and psychological process mediators; b) process mediators affect healthy lifestyle decay curve. If all conditions are met, the test of mediation will be whether behavioral and psychological process mediators fully or partially explain the effect of condition on relapse curves.

### Sample Size and Power

The effect size calculations assume a two-sided alpha set at .05 and were estimated for a power of .85.

#### Primary Aim: Healthy Lifestyle

The outcomes for the primary aim are the z-scores for fruit and vegetables (FV), saturated fat (Fat), physical activity (PA) and sedentary activity (Sed) separately and averaged to form the composite healthy lifestyle score. Using pilot data to compare any one treatment condition to the other three, we found average effect sizes equal to .66, .63, .21 and .47 for change in FV, PA, Sed, and Fat, respectively. Therefore, we anticipate that we will have effect sizes approximately equal to .5 for the three comparisons on each outcome variable. Given an effect size of .5, a total of 200 participants, 50 per group, will yield adequate power for testing the three contrasts.

#### Secondary Aim: Behavioral and Psychological Processes

Whether participants adhere to each prescription can be verified by consumption elasticity: the degree to which each targeted behavior changes in response to prescription. Again using pilot data, we compared the target behaviors' elasticity for any one condition to the average elasticity of target behaviors for the other three conditions and found a mean difference of .437 (sd = .25) in elasticity, which yields an effect size of 1.78 to detect differences in adherence between one condition and all others. Given the effect size, our projected sample size of 50 per group should be adequate to detect differences in adherence across the planned contrasts. Our hypotheses also lead us to be interested in detecting differences between prescriptions in the magnitude of healthy substitution. Based on pilot data, we found a mean difference of .11 (sd = .08) in substitutability when we compared any one condition to the other three, which yields an effect size of 1.46. For an effect size of 1.46 a total of 50 subjects should suffice to test the contrasts.

Relevant outcomes are mood, craving for unhealthy foods and activities, and attentional allocation toward them. Mood will be measured daily during baseline and prescription phases, while craving and attentional allocation to unhealthy temptations will be measured during the two laboratory sessions. Based on pilot data, we found mean differences of 2.108 (sd = 6.057) for POMS vigor and 3.627 (sd = 10.275) for POMS dysphoria when we compared any one group to the other three. Based on the effect size estimates of .34 on vigor and .35 on dysphoria a sample size of 50 participants per group should yield adequate power.

## Discussion

Considerable evidence links poor quality diet and physical inactivity not only to adverse health consequences [1-3] but also to each other. That is, poor quality diet and physical inactivity are characterized by a pattern of co-occurrence or multi-behavioral morbidity [9-11]. Being able to reduce bundled risk behaviors simultaneously would create the potential to do greater good, in less time, with less effort, and at less cost. The ability to efficiently capitalize on teachable moments for behavior change would be advantageous for health care providers, patients, and policy makers. Yet, despite the strong appeal of multiple behavior change interventions, a paucity of research demonstrates their feasibility let alone their success. The Make Better Choices study was designed both to address the feasibility of changing diet and activity behaviors simultaneously, and to identify the optimal combination of goals to maximize overall behavior change.

Here we have described the Make Better Choices design and procedure. The MBC RCT will randomize 200 sedentary adults with poor quality diet to improve one category of unhealthy dietary behavior and one category of unhealthy physical activity behavior simultaneously. Participants will be prescribed one of four interventions that recommend all possible combinations of increasing healthy and decreasing unhealthy diet and activity options. Healthful change will be assessed for both targeted and untargeted (collateral) behavioral outcomes.

This design will permit us to evaluate three alternative hypotheses: 1) *familiarity*, which predicts best outcome from prescribing usual dieting behaviors (↓FAT ↑PA);2) *behavioral economic*, which predicts best outcome from the condition that maximizes substitutive and complementary healthy change in both targeted and untargeted behaviors(↑FV ↓Sed); and 3) *low inhibitory demand *, which predicts best outcome from the condition that minimizes inhibitory demands (↑FV↑PA).

The mechanisms that underlie successful simultaneous change in health risk behavior bundles remain poorly understood. An important, novel feature of this research is the examination of change in collateral behaviors not targeted directly by an intervention. Examination of effects on untargeted outcomes is usually undertaken in order to identify unintended adverse consequences of an intervention. Here, instead, we examine whether untargeted healthy behavior changes can be harnessed effortlessly and to advantage.

Another key innovation in MBC involves the evaluation of a handheld PDA decision support tool that facilitates self-monitoring and remote communication with a behavioral coach. The use of technology that supports in vivo self-monitoring and timely distance coaching affords a potentially scalable, cost effective way to support health behavior change.

Limitations on the generalizability of study results warrant consideration. The requirement that enrollees persist in displaying all four risk behaviors despite throughout a baseline period will likely bias the sample towards those with severe multi-behavioral morbidity. Whether outcomes would differ in those with less recalcitrant or pervasive lifestyle risk behaviors warrants examination. Further, because MBC is designed to examine the feasibility and success of a new intervention under ideal conditions, participants will be paid to participate. It cannot be assumed that comparable outcomes would result in the absence of financial incentives.

In sum, the MBC study represents an important step toward identifying and improving strategies to address bundled health risk behaviors. The trial will permit hypothesis testing about optimal intervention to promote simultaneous diet and activity change. The high prevalence of health risk behaviors, the human and financial costs associated with chronic disease, and our poor understanding of how to reduce risk behaviors most efficiently all suggest the importance of the study.

## Competing interests

The authors declare that they have no competing interests.

## Authors' contributions

BS, the principal investigator, was responsible for study conceptualization and design. She collaborated with KS, DH, and LE on developing the analytic plan. MS and JV participated in operationalizing the intervention. HGM developed the data management plan. BS, ATK, JV, ACM, HGM, SWR, and AD drafted the manuscript. All authors read and approved the final manuscript.

## Pre-publication history

The pre-publication history for this paper can be accessed here:

http://www.biomedcentral.com/1471-2458/10/586/prepub
